# Performance of a core of transversal skills: self-perceptions of undergraduate medical students

**DOI:** 10.1186/s12909-016-0527-2

**Published:** 2016-01-15

**Authors:** Laura Ribeiro, Milton Severo, Maria Amélia Ferreira

**Affiliations:** Department of Medical Education and Simulation, Faculty of Medicine, University of Porto, Al. Prof. Hernâni Monteiro, 4200-319 Porto, Portugal; Department of Biochemistry, Faculty of Medicine, University of Porto, Al. Prof. Hernâni Monteiro, 4200-319 Porto, Portugal; Instituto de Investigação e Inovação em Saúde (I3S), Universidade do Porto, Porto, Portugal; Department of Clinical Epidemiology, Predictive Medicine and Public Health, Faculty of Medicine, University of Porto, Al. Prof. Hernâni Monteiro, 4200-319 Porto, Portugal

**Keywords:** Transversal skills, Undergraduate, Medical students

## Abstract

**Background:**

There is an increasingly growing trend towards integrating scientific research training into undergraduate medical education. Communication, research and organisational/learning skills are core competences acquired by scientific research activity. The aim of this study was to assess the perceived performance of a core of transversal skills, related with scientific research, by Portuguese medical students.

**Methods:**

A cross-sectional study was conducted in 611 Portuguese students attending the first, fourth and sixth years of the medical course, during the same academic year. A validated questionnaire was applied for this purpose.

**Results:**

Medical students felt confident regarding the majority of the analyzed transversal skills, particularly regarding team work capacity (72.7 % perceived their own capacity as good). On the other hand, the perceived ability to manage information technology, time and to search literature was classified only as sufficient by many of them. The progression over the medical course and participation in research activities were associated with an increasing odds of a good perceived performance of skills such as writing skills (research activity: OR = 2.00; 95 % CI: 1.34–2.97) and English proficiency (research activity: OR = 1.59; 95 % CI: 1.06–2.38/final year medical students: OR = 3.63; 95 % CI: 2.42–5.45).

**Conclusions:**

In this line, the early exposure to research activities along undergraduate medical education is an added value for students and the implementation of an integrated research program on medical curriculum should be considered.

**Electronic supplementary material:**

The online version of this article (doi:10.1186/s12909-016-0527-2) contains supplementary material, which is available to authorized users.

## Background

In the 90s of the last century, following the developments in the USA, several Western European universities responded to the need looking for research capacity beyond medical students [1]. Medical doctors are the first to experience the need for more evidence-based knowledge in order to deliver better patient care. However, their capability to participate in medical research programs, and to inspire them, is often limited by both lack of training during their studies and the reduced amount of time available for research, even in university hospitals [1]. Consequently, there is an increasingly growing trend towards integrating scientific research training into undergraduate medical education [1–3].

Medical curriculum in Europe follows the quality assurance frameworks and the portability of qualifications of the directives of Bologna Process [4]. In Portugal, the medical course was reformulated into an Integrated Master Course in Medicine and is currently composed by a first cycle of three years, corresponding to a degree in basic health sciences, and a second cycle also lasting three years of clinical learning and professional clerkship.

At the Faculty of Medicine of the University of Porto (FMUP), along the first cycle of studies all students participate in random research activities in curricular units such as biochemistry, cellular biology and genetics, and some participate in research projects as extra-curricular activities, usually since the fourth year.

Scientific research involves the collection and processing of new information and its interpretation through logic and previous knowledge and experience [3]. Critical thinking, communication ability, literature searching, cope with information technology, team work, solving-problems and self-improving learning are scientific skills acquired through research activity [5–7]. Some of these skills are required for effective decision-making being a valuable quality required in any program in higher education [8, 9]. However, transversal skills of medical freshman students, as well as their development throughout the medical course, have not yet been assessed, especially considering the recent reforms in medical education. Our research group has been studying several issues related with undergraduate medical education [10–12].

The aim of this study, again reflecting our concerns on this matter, was to assess the perceived performance of a core of transversal skills by undergraduate medical students.

## Methods

The specific objectives of this study were: (1) To evaluate if self-perception of medical students about transversal skills execution change over the progression of the medical course and (2) To compare the above objective by gender, type of school attended at high schooling level (public or private) and previous involvement (or not) in research projects.

A cross-sectional study was conducted in order to develop this research. Among the 796 eligible medical students, 611 (76.8 %) have participated in the study. The participants were enrolled in the Master Degree Course in Medicine of FMUP, Portugal. The study included freshman students (first year), those who concluded the first cycle of studies (beginning of the fourth year) and students who completed the entire medical course (end of the sixth year). All students from the first and fourth years answered the questionnaire at once at the beginning, and those from the six year at the end, of the same academic year.

Medical students were invited to fulfill an anonymous self-administered and validated questionnaire: “Importance of Transversal Skills for Clinical Practice” (ITS4CP) developed by the authors [12]. The transversal skills included in the questionnaire were adapted from AMEE Medical Education Guide [3].

'The perceived ability to perform the transversal skills was assessed through this questionnaire, following the Likert scale: 1 (good), 2 (sufficient), 3 (insufficient) and 4 (poor). This scale showed a good reliability and validity: the reliability was high for the 11 skills and the three factors described below, and the construct validity was set by the Confirmatory Factor Analysis (CFA). The 11 transversal skills were grouped into three factors: communication skills (CS), research skills (RS) and organisational/learning skills (OS). The CS factor included writing communication (WC), oral communication (OC) and visual communication (VC). The RS factor included the ability to perform literature searching (LS), cope with information technology (IT) and data analysis (DA) and English proficiency (EP). The OS factor included team work (TW) capacity, time management (TM), the ability to solve problems (SP) and to self-improve learning (IL). CFA was used to test the fit of the model with 3-factor structure considering the 11 skills as items. CFA fit was assessed using different indexes: (i) the Tucker–Lewis Index (TLI) [13], (ii) the Comparative fit index (CFI) [14], (iii) the Route mean square error of approximation (RMSEA) [15], and (iv) the Standardized root mean square residual (SRMR) [16]. A good model fit is indicated by a CFI and TLI values of 0.90 or higher [16] and values of RMSEA and SRMR close to 0 [17]. The internal reliability of the scale was tested using the Cronbach’s alpha coefficient.

The perceived ability to perform the transversal skills was recoded, in comparison with the scale shown in Additional file 1, in two categories sufficient (0) vs. good (1), given that no student perceived his/her own ability as insufficient or poor. The percentage of medical students who perceived their performance as good was estimated. Odds ratio (OR) was used to estimate the magnitude of the association between the perceived ability to perform each transversal skill and several factors (gender, academic year, participation in research projects and high school). Unconditional logistic regression adjusted for all variables was used to estimate the OR and the respective 95 % confidence interval. All statistics analyses were performed with the R 2.12.1 [18].

The ethical principles of this research followed the guidelines approved by the Ethics Committee of FMUP.

## Results

**Fig. 1:**
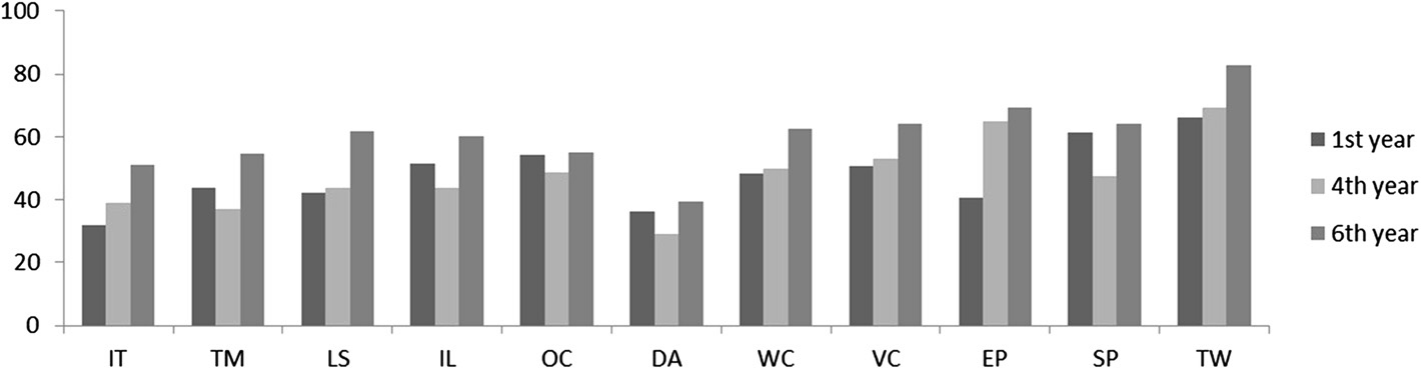
Confirmatory Factor Analysis for three-factor model of the Transversal Skills scale. Legend: Factor CS represents communication skills, Factor RS represents research skills and Factor OS Organizational/learning Skills. Model Chisquare = 142.67 Df = 41 Pr(>Chisq) <0.001, RMSEA index = 0.062591 90 % CI: (0.051578, 0.073944), Bentler-Bonnett NFI = 0.95052, Tucker-Lewis NNFI = 0.95178, Bentler CFI = 0.96405, SRMR = 0.033019

Characteristics of the sample are summarized in Table 1. The Cronbach’s alpha of the analysis of the 11 transversal skills ranges from 0.651 to 0.818 indicating a moderate to high internal consistency. According to the theoretical model, in the CFA it was assumed that items WC, OC, and VC belong to factor CS (Communication Skills), items LS, IT, DA and EP belong to factor RS (Research Skills), and items TW, TM, SP and IL belong to factor OS (Organizational/learning Skills) and they were correlated with each other. Figure 1 shows the factor loadings supporting these relations. The model global fitness was tested and confirmed. The Cronbach alpha was 0.69, 0.81, 0.80 for factor CS, RS and OS, respectively.

**Table 1:** Characteristics of the sample

	*n* (%)
Gender	
Female	397 (65.0)
Male	214 (35.0)
Academic year	
1^st^ year	251 (41.1)
4^th^ year	148 (24.2)
6^th^ year	212 (34.7)
Participation in research activities	
No	460 (75.3)
Yes	151 (24.7)
High school	
Public	442 (72.3)
Private	169 (27.7)

**Fig. 2:**
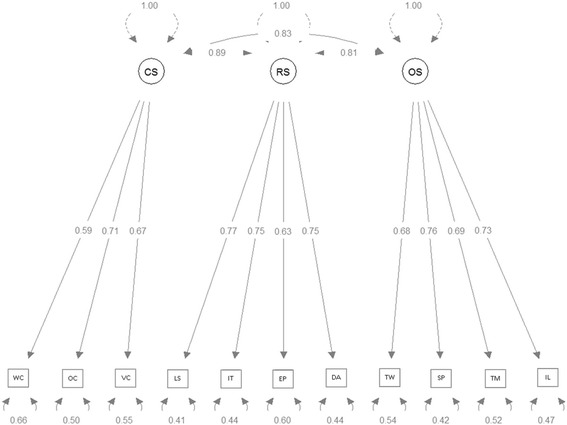
Percentage of medical students who perceived their performance as good, according to the corresponding transversal skills. Legend: IT, information technology; TM, time management; LS, literature searching; IL, improving learning; OC, oral communication; DA, data analysis; WC, writing communication; VC, visual communication; EP, English proficiency; SP, solving problems; TW, team work

No student perceived his/her own ability to perform the transversal skills as insufficient or poor. More than 50 % of students considered their own ability to manage information technology, of time management and to perform literature searching as sufficient. In relation to the other transversal skills, more than 50 % of students considered their own ability to perform them as good (Fig. 2). 72.7 % of medical students perceived their own capacity of team work as good (Fig. 2).

The progression over the medical course, as well as the participation in research activities, was associated with an increasing odds of a good perceived performance of communication skills (Table 2). Regarding writing skills, an approximate 2-fold increase in odds of a good performance was observed in sixth year students and in those who participated in research activities (Table 2).

**Table 2:** Factors associated with communication skills performance

		Communication skills, OR (95 % CI)				
		Writing		Oral		Visual
	*n* (%)	OR (95 % CI)	*n* (%)	OR (95 % CI)	*n* (%)	OR (95 % CI)
Gender						
Female	67 (16.9)	1	119 (30.1)	1	47 (11.9)	1
Male	21 (9.7)	0.78 (0.55–1.11)	42 (19.5)	1.09 (0.78–1.54)	21 (10.0)	0.98 (0.69–1.38)
Academic year						
1^st^ year	25 (10.0)	1	42 (16.9)	1	15 (6.1)	1
4^th^ year	17 (11.4)	1.14 (0.75–1.74)	39 (26.4)	0.79 (0.52–1.19)	16 (11.0)	1.08 (0.71–1.64)
6^th^ year	46 (21.6)	2.11 (1.42–3.13)	80 (37.6)	1.18 (0.81–1.73)	37 (17.5)	1.76 (1.19–2.60)
Participation in research activities						
No	64 (14.0)	1	121 (26.6)	1	41 (9.1)	1
Yes	24 (15.7)	2.00 (1.34–2.97)	40 (26.1)	1.36 (0.93–1.99)	25 (16.6)	1.38 (0.94–2.02)
High school						
Public	65 (14.8)	1	114 (26.1)	1	44 (10.1)	1
Private	16 (10.7)	0.79 (0.54–1.17)	36 (24.0)	1.15 (0.78–1.68)	17 (11.6)	1.32 (0.89–1.93)

Adjusted for all variables in the table

The progression over the medical course and the participation in research activities were also associated with an increasing odd of a good perceived performance of research skills and organisational/learning skills (Tables 3 and 4). More than 3-fold increase in odds of a good perceived English proficiency was observed in the students of the sixth year of medical course (Table 3).

**Table 3:** Factors associated with research skills performance

		Research skills						
		Literature searching		Information technology		Data analysis		English proficiency
	*n* (%)	OR (95 % CI)	*n* (%)	OR (95 % CI)	*n* (%)	OR (95 % CI)	*n* (%)	OR (95 % CI)
Gender								
Female	113 (28.8)	1	76 (19.4)	1	77 (19.5)	1	125 (31.9)	1
Male	55 (25.6)	0.99 (0.70–1.40)	31 (14.1)	2.20 (1.55–3.14)	40 (18.4)	1.77 (1.24–2.52)	57 (26.4)	1.43 (0.99–2.05)
Academic year								
1^st^ year	25 (10.0)	1	29 (11.7)	1	30 (12.0)	1	41 (16.5)	1
4^th^ year	61 (41.8)	1.07 (0.70–1.63)	24 (16.4)	1.31 (0.84–2.05)	29 (19.7)	0.71 (0.45–1.11)	50 (34.2)	2.74 (1.77–4.24)
6^th^ year	82 (38.7)	2.29 (1.55–3.38)	54 (25.4)	2.32 (1.56–3.46)	58 (27.2)	1.22 (0.82–1.81)	91 (42.7)	3.63 (2.42–5.45)
Participation in research activities								
No	118 (26.0)	1	72 (15.9)	1	84 (18.5)	1	135 (29.9)	1
Yes	48 (31.8)	1.69 (1.15–2.48)	34 (22.4)	1.23 (0.83–1.83)	31 (20.3)	1.30 (0.87–1.92)	46 (30.1)	1.59 (1.06–2.38)
High school								
Public	121 (27.7)	1	77 (17.7)	1	85 (19.4)	1	128 (29.3)	1
Private	36 (24.3)	1.15 (0.78–1.69)	23 (15.4)	0.89 (0.59–1.32)	28 (18.8)	1.18 (0.80–1.75)	42 (28.6)	1.13 (0.76–1.68)

Adjusted for all variables in the table

**Table 4:** Factors associated with organisational/learning skills performance

		Organisational/learning skills						
		Team work		Time management		Solving problems		Improving learning
	*n* (%)	OR (95 % CI)	*n* (%)	OR (95 % CI)	*n* (%)	OR (95 % CI)	*n* (%)	OR (95 % CI)
Gender								
Female	153 (38.8)	1	172 (43.4)	1	165 (41.7)	1	154 (38.9)	1
Male	65 (30.4)	0.72 (0.49–1.05)	84 (39.3)	0.84 (0.59–1.18)	80 (37.0)	0.97 (0.68–1.37)	75 (34.7)	1.19 (0.84–1.68)
Academic year								
1^st^ year	62 (24.9)	1	68 (27.2)	1	69 (27.6)	1	53 (21.2)	1
4^th^ year	56 (38.1)	1.10 (0.71–1.72)	77 (52.0)	0.69 (0.45–1.06)	67 (45.3)	0.56 (0.37–0.85)	60 (40.5)	0.73 (0.48–1.11)
6^th^ year	100 (47.2)	2.60 (1.63–4.13)	111 (52.4)	1.59 (1.08–2.33)	109 (50.9)	1.16 (0.79–1.72)	116 (54.2)	1.54 (1.05–2.27)
Participation in research activities								
No	163 (36.0)	1	184 (40.5)	1	187 (40.9)	1	167 (36.6)	1
Yes	53 (34.9)	1.00 (0.66–1.52)	69 (45.1)	1.45 (0.99–2.13)	56 (36.8)	1.14 (0.77–1.68)	60 (39.2)	1.69 (1.14–2.48)
High school								
Public	150 (34.5)	1	188 (43.0)	1	173 (39.5)	1	165 (37.7)	1
Private	55 (36.9)	1.16 (0.75–1.78)	56 (37.6)	0.76 (0.52–1.12)	60 (40.0)	1.17 (0.79–1.73)	55 (36.7)	1.19 (0.84–1.68)

Adjusted for all variables in the table

Male students were associated with an increasing odds of a good perceived manage of information technology and a good performance of data analysis, comparatively to female students (Table 3).

Attending a private school at high school was associated with an increasing odds of a good performance in the majority of skills, although without statistical significance (Tables 2, 3 and 4).

## Discussion

This group of medical students felt confident regarding the majority of the studied transversal skills. The progression along the medical course, as well as the participation in research activities might have contributed to this perception. These findings are in line with what has already been investigated regarding the improvement of transversal skills due to the engagement in research activities [5–7].

On the other hand, the perceived ability to manage information technology, time management and to perform literature searching was classified as sufficient by many of the students. Therefore, improvements in medical education should be further explored in order to develop these competences.

Regarding the information technology, medical educators are rapidly integrating technology into the medical curricula as a necessary intervention [19, 20]. According to Ranasinghe et al., there is a need to improve computer literacy among freshman medical students, by increasing computer training in schools, or by introducing computer training in the initial stages of the medical course [21]. The study by Lim et al. showed that 81.5 % of final year medical students consider their computer skills adequate, despite additional steps need to be taken to increase the use of Internet as a method of instruction [22]. Several studies have explored the incorporation of personal digital assistants (PDAs) [23, 24], e-learning modalities [25] and social networking for interprofessional instruction [26] in medical education.

In order to avoid the workload that medical students usually face, many schools are implementing policies to restrict student work hours [27]. In line with this workload, our students also had difficulties in time management.

Literature searching is an essential competence for both research and clinical activities, and our students could benefit and feel more confident with specific training on this skill. A literature review examining the effectiveness of literature searching skills instruction for medical students showed that although the current instructional methods are effective, there is little evidence that learning persists over time [28].

On the other hand, a significant number of medical students perceived their own team work capacity as good. This ability should continue to be explored during the medical course, for instance through students’ involvement in small research groups. Indeed, teamwork has become a major focus in healthcare [29], partially, in consequence of the report provided by the Institute of Medicine, which details the high rate of preventable medical errors, mainly originated from dysfunctional or nonexistent teamwork [30].

In relation to the putative determinants of perceptions about transversal skills, students who participated in research projects revealed an increased odd of good perceived performance in all transversal skills, including those mentioned above. In this analysis, writing ability stood out between communication skills. In health professions education, writing has been used as a tool to promote a variety of learning goals, but students in medical professions often lack the required writing skills during their education and career [31, 32]. Participation in research activities is therefore essential since students who write concisely and logically, use terminology accurately and justify their assertions, show better academic performance [31]. Furthermore, scientific writing for publication is competitive and demanding for researchers [33], and training in writing skills along the medical course is associated with a significant improvement in text quality, structure and argumentation [34].

Students who finished the medical course demonstrated a significant increase of odds to have a good perceived English proficiency. Probably, this is explained by the need to use international scientific literature during the study years in medical school. However, students should feel confident in their English proficiency from the beginning of the course in order to have an efficient acquisition of knowledge. For the novice English-as-a-second-language researcher, the pressure to publish compounds the difficulties of mastering the English language [33]. In addition, in most countries around the world, local medical students outperform international students, in an academic sense, probably because of the acquision of language proficiency skills [35]. According to Hays et al. Australian medical students whose primary language is other than English, yet with poor English oral communication skills, should be encouraged to speak English away from the medical school and should be offered additional tuition so that their skills in other languages are not lost to the health-care system [36]. On the other hand, Mirza et al. reported that communication skills training purely in English can leave Arab medical students ill equipped to communicate with patients in their own communities and tongue [37].

In relation to problem solving skills, fourth year students demonstrated a significant decrease in the odds of good perceived performance, contrary to freshman and final year students. Problem solving skills has been defined as a hypothetical-deductive activity engaged by physicians, in which the early generation of hypotheses influences the subsequent gathering of information [38]. In the fourth year of the medical course, students begin the clinical practice, hence their perception of a poorer performance can be, partially, explained by the unfulfilled expectations during the first cycle of studies, as well as the fear of becoming incompetent practitioners. Therefore, the need to include instructional activities to promote the development of problem-solving abilities should be asserted in the medical curriculum.

Concerning gender, male students were associated with an increasing odds of a good perceived management of information technology, comparatively to female students. This finding has been reported by some authors who showed that females had more negative attitudes towards computers and Internet than males [39, 40]. Comparison at high schooling level between private and public schools, showed no significant differences. Nonetheless, further studies are needed to explore whether private schools offer a better support for students’ research competences than public schools.

This study assessed the putative performance of a core of transversal skills by Portuguese medical students based on their perceptions. An increased body of evidence shows that student perceptions about several aspects of their competences can be valuable for extracting evaluative conclusions regarding the quality of medical education [41, 42], despite this approach could be seen as a study limitation.

No student perceived his own ability to execute the transversal skills as insufficient or poor, a finding that may reflect a limitation of the used Likert scale, that require to be revised in future studies. This study has the inherent limitations of a cross-sectional study. Thus, a follow-up study of these students’ cohorts could also provide additional information of their transversal skills’ perceptions, as well as important data for the design of a medical educational program.

## Conclusions

In conclusion, this group of medical students felt confident regarding the majority of the studied skills, particularly team work capacity. However, conceptual models of medical education modalities should be further explored to improve skills, such as both information technology and time management as well as literature searching.

The progression over the medical course and participation in research activities were associated with an increased odd of a good perceived performance of all transversal skills. Thus, the early exposure to research activities along undergraduate medical education is an added value for students and the implementation of an innovative integrated research program into the medical curriculum should be considered.

## Additional file

Additional file 1:
**Questionnaire**
