# Supplementary material for: Performance of a core of transversal skills: self-perceptions of undergraduate medical students
Source: BMC Med Educ. 2016 Jan 15;16:18. doi: 10.1186/s12909-016-0527-2 (PMC4715346; doi:10.1186/s12909-016-0527-2)
Supplement: Additional file 1: — Questionnaire [file 12909_2016_527_MOESM1_ESM.docx]

**Appendix 1 - Questionnaire**

| **Scientific skills** | | **Writing communication** | **Oral communication** | **Visual communication** | **Literature searching** | **Information technologies** | **English proficiency** | **Data analysis** | **Team-work** | **Solving-problems** | **Time management/ self-organization** | **Improving learning** |
| --- | --- | --- | --- | --- | --- | --- | --- | --- | --- | --- | --- | --- |
| **1.** Importance during the high school | Very important |  |  |  |  |  |  |  |  |  |  |  |
|  | Important |  |  |  |  |  |  |  |  |  |  |  |
|  | Not very important |  |  |  |  |  |  |  |  |  |  |  |
|  | Unimportant |  |  |  |  |  |  |  |  |  |  |  |
| **2.** Importance for assessment at high school | Very important |  |  |  |  |  |  |  |  |  |  |  |
|  | Important |  |  |  |  |  |  |  |  |  |  |  |
|  | Not very important |  |  |  |  |  |  |  |  |  |  |  |
|  | Unimportant |  |  |  |  |  |  |  |  |  |  |  |
| **3.** Importance for clinical practice | Very important |  |  |  |  |  |  |  |  |  |  |  |
|  | Important |  |  |  |  |  |  |  |  |  |  |  |
|  | Not very important |  |  |  |  |  |  |  |  |  |  |  |
|  | Unimportant |  |  |  |  |  |  |  |  |  |  |  |
| **4.** Personal importance of this skill for you | Very important |  |  |  |  |  |  |  |  |  |  |  |
|  | Important |  |  |  |  |  |  |  |  |  |  |  |
|  | Not very important |  |  |  |  |  |  |  |  |  |  |  |
|  | Unimportant |  |  |  |  |  |  |  |  |  |  |  |
| **5.** How do you score your ability to execute it? | Good |  |  |  |  |  |  |  |  |  |  |  |
|  | Sufficient |  |  |  |  |  |  |  |  |  |  |  |
|  | Insufficient |  |  |  |  |  |  |  |  |  |  |  |
|  | Poor |  |  |  |  |  |  |  |  |  |  |  |
